# **INO1** transcriptional memory leads to DNA zip code-dependent interchromosomal clustering

**DOI:** 10.15698/mic2015.12.242

**Published:** 2015-11-13

**Authors:** Donna G. Brickner, Robert Coukos, Jason H. Brickner

**Affiliations:** 1 Department of Molecular Biosciences, Northwestern University, Evanston, IL USA 60201.

**Keywords:** epigenetic inheritance, transcriptional memory, nuclear pore, DNA zip code, interchromosomal clustering

## Abstract

Many genes localize at the nuclear periphery through physical interaction with the nuclear pore complex (NPC). We have found that the yeast *INO1* gene is targeted to the NPC both upon activation and for several generations after repression, a phenomenon called epigenetic transcriptional memory. Targeting of *INO1* to the NPC requires distinct *cis-*acting promoter DNA zip codes under activating conditions and under memory conditions. When at the nuclear periphery, active *INO1* clusters with itself and with other genes that share the GRS I zip code. Here, we show that during memory, the two alleles of *INO1* cluster in diploids and endogenous *INO1* clusters with an ectopic *INO1* in haploids. After repression, *INO1* does not cluster with GRS I - containing genes. Furthermore, clustering during memory requires Nup100 and two sets of DNA zip codes, those that target *INO1* to the periphery when active and those that target it to the periphery after repression. Therefore, the interchromosomal clustering of *INO1* that occurs during transcriptional memory is dependent upon, but mechanistically distinct from, the clustering of active *INO1*. Finally, while localization to the nuclear periphery is not regulated through the cell cycle during memory, clustering of *INO1* during memory is regulated through the cell cycle.

## INTRODUCTION

Eukaryotic genomes are spatially organized. Chromosomes compact, form intrachromosomal loops and interact with each other and with subnuclear structures 1. Such interactions lead to stereotypical arrangements of chromosomes with respect to each other and with respect to nuclear landmarks.

Individual genes often change their position when induced or repressed. A well-studied phenomenon that illustrates this point and that serves as an excellent model is the movement of yeast genes from the nucleoplasm to the nuclear periphery upon activation 2,3. Inducible genes such as *INO1*, *GAL1-10*, *GAL2, TSA2*, *HSP104* and *HXT1* move to the nuclear periphery and physically interact with the NPC upon activation 2,3,4,5,6,7. Mutations in nuclear pore proteins (Nups) block targeting to the periphery 6,8,9,10 and genome-wide ChIP experiments in yeast, flies, and mammalian cells indicates that hundreds to thousands of genes interact with NPCs or nuclear pore proteins 3,11,12,13,14. Thus, interaction with the NPC leads to changes in gene positioning.

**Figure 1:**
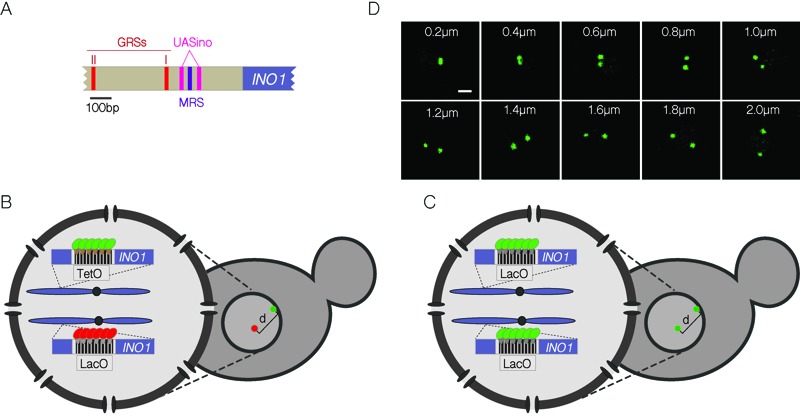
FIGURE 1: Experimental system. **(A) **Schematic of the *INO1* promoter, with the relevant regulatory elements and DNA zip codes highlighted. GRS: Gene Recruitment Sequence 6; MRS: Memory Recruitment Sequence 10; UAS_INO_: Upstream Activating Sequence regulated by inositol. **(B and C) **Experimental setups for studying interchromosomal clustering using two different repressor arrays **(B)** or two identical arrays **(C)**. **(D)** Representative confocal micrographs of cells having two GFP-marked arrays. Scale bar = 1µm.

Interaction of yeast genes with the NPC and positioning to the nuclear periphery requires small *cis*-acting DNA elements in their promoters 6,7,10. For example, two elements called GRS I and GRS II in the *INO1* promoter are necessary for targeting to the NPC (Figure 1A; ref. 6). These elements function as *DNA zip codes*: they are both necessary for *INO1 *targeting and, when inserted at an ectopic site in the genome, they are sufficient to induce repositioning to the nuclear periphery and interaction with the NPC. The GRS I element binds to the Put3 transcription factor, which is required for GRS I-mediated positioning 7. Thus, genomes *encode* subnuclear positioning through transcription factor binding sites that function as DNA zip codes.

In addition to promoting interaction with the NPC, DNA zip codes like GRS I promote interchromosomal clustering of genes 7. The two alleles of *INO1* in diploid cells cluster together upon activation. In haploids, *INO1* clusters with another GRS I-targeted gene, *TSA2* and with GRS I inserted at an ectopic locus 7. Mutations in GRS I, loss of Nups or loss of Put3 disrupt interchromosomal clustering. Therefore, DNA zip codes such as the GRS I, are necessary and sufficient to induce interchromosomal clustering of active loci through interaction with transcription factors and the NPC.

Upon repression, the *INO1* gene remains associated with the NPC for several generations, a phenomenon called epigenetic transcriptional memory 8. This interaction involves a different *cis*-acting DNA zip code (the Memory Recruitment Sequence, MRS; Figure 1A) and different nuclear pore proteins (e.g. Nup100; refs 10, 15). Mutations in the GRS I and II elements do not affect targeting to the NPC during memory and mutations in the MRS do not affect targeting to the NPC during activation, suggesting that these two mechanisms are independent 8. Here, we show that transcriptional memory also leads to interchromosomal clustering*. *Clustering during memory requires both previous clustering of active *INO1* and the MRS zip code. However, unlike under activating conditions, *INO1 *does not cluster with the GRS I at an ectopic site under memory conditions. Therefore, *INO1 *clusters with different partners under activating and memory conditions, suggesting that interchromosomal clusters are remodeled upon repression. Finally, unlike targeting to the periphery 16, *INO1 *clustering is regulated through the cell cycle under both activating and memory conditions. These results show that interchromosomal clustering of *INO1* during transcriptional memory is zip code-dependent but represents a molecular event that is distinct from targeting to the NPC.

## RESULTS

### *INO1* clustering during transcriptional memory

To monitor clustering of *INO1*, we utilized several experimental systems: diploid strains in which one allele of *INO1* is marked with an array of 128 Lac Operator (LacO) repeats and the other allele is marked with an array of 112 Tet Operator (TetO) repeats (Figure 1B), diploid strains in which both alleles of *INO1* are marked with LacO (Figure 1C) or haploid strains in which *INO1* is marked with TetO and other sites (i.e. *URA3* or *GAL1*) are marked with LacO (similar to the system shown in Figure 1B). The distance between the two genes can be measured in each cell in a population (Figure 1D) and clustering can be assessed by comparing the distribution of distances between the two loci in the population or by measuring the fraction of cells in which the two loci are ≤ 0.55 µm apart 7.

**Figure 2:**
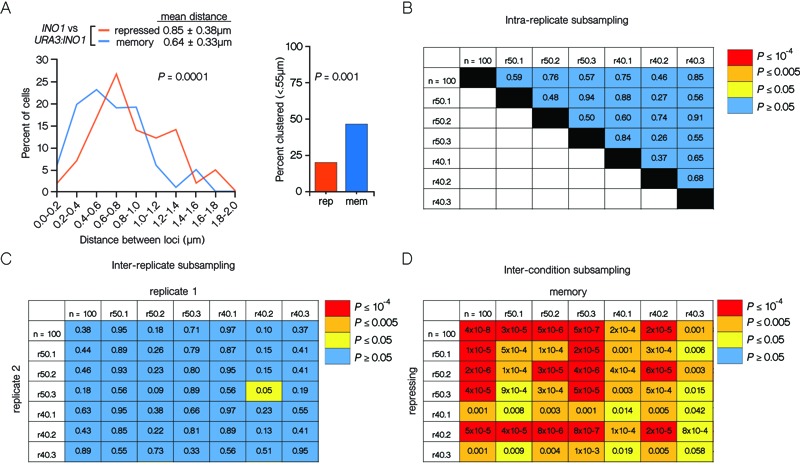
FIGURE 2: *INO1* transcriptional memory leads to interchromosomal clustering. **(A) **Haploid cells having the endogenous *INO1 *gene marked with the TetO and *URA3:INO1* marked with the LacO, expressing GFP-TetR and mRFP-LacI 8 were grown under *INO1 *repressing (+ inositol) or memory (- inositol → + inositol, 3h) conditions, fixed and processed for immunofluorescence against GFP and mRFP. **Left:** The distribution of distances between the two loci in ~100 cells, binned into 0.2 µm bins. *P* values were calculated using a Wilcoxon Rank Sum Test. **Right:** the fraction of cells in which the two loci were ≤ 0.55 µm. *P* values were calculated using a Fisher Exact Test. Note: the distribution of the repressed condition has been previously published 7 and is shown only for comparison to the distribution under the experimental (memory) condition. **(B-D)** Subsampling analysis. Full datasets (n = 100) or randomly generated subsamples of 50 or 40 measurements (r50 or r40, respectively) were compared pairwise using a Wilcoxon Rank Sum test. The numbers in each cell are the *P* values, color-coded as described in the legend. **(B) **A biological replicate compared with itself. **(C)** Two biological replicates compared with each other. **(D) **Distributions from repressing and memory conditions compared with each other.

**Figure 3:**
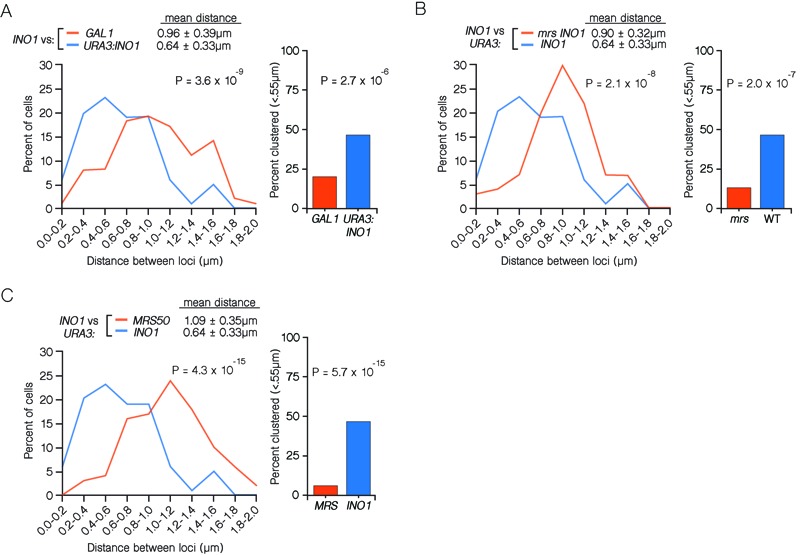
FIGURE 3: *INO1* interchromosomal clustering during memory is specific and MRS-dependent. **(A-C)** Haploid cells having the endogenous *INO1 *gene marked with the TetO and either *URA3* **(A-C)** or *GAL1 ***(A) **marked with the LacO, expressing GFP-TetR and mRFP-LacI 8 were grown under *INO1 *memory (activating → repressing, 3h) conditions, fixed and processed for immunofluorescence against GFP and mRFP. **Left:** The distribution of distances between the two loci in ~ 100 cells, binned into 0.2 µm bins. *P* values were calculated using a Wilcoxon Rank Sum Test. Right: the fraction of cells in which the two loci were ≤ 0.55 µm. *P* values were calculated using a Fisher Exact Test. Note that the data used to generate the distribution for the control of *INO1-TetO* vs. *URA3:INO1-LacO* is the same in all three panels. The combinations tested were *INO1-TetO* vs. *URA3:INO1-LacO* **(A-C)**, or *INO1-TetO *vs. *GAL1-LacO* **(A),** *INO1-TetO* vs. *URA3:mrsINO1-LacO* **(B)** and *INO1-TetO* vs. *URA3:MRS50-LacO* **(C)**.

**Figure 4:**
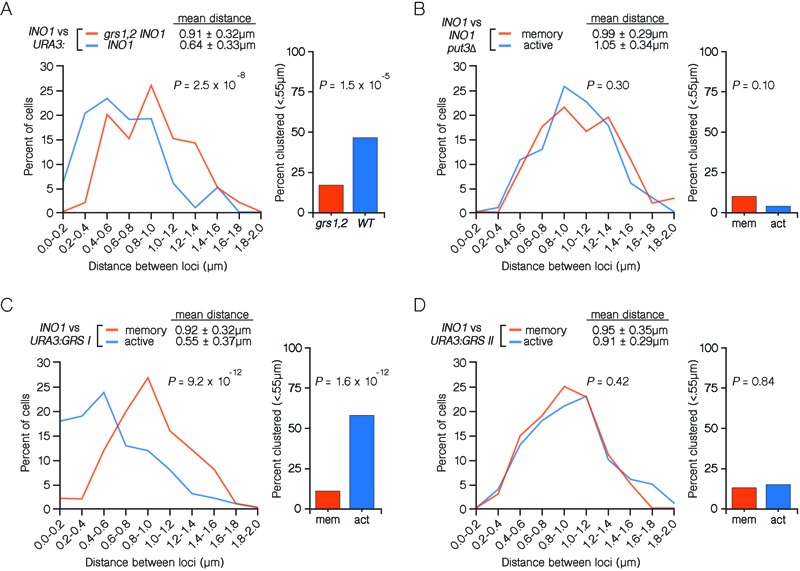
FIGURE 4: *INO1* interchromosomal clustering during transcriptional memory requires clustering of active *INO1*. **(A and B) **Haploid cells having the LacO array integrated at *URA3* and the TetO array integrated at *INO1,* and expressing GFP-TetR and mRFP-LacI were fixed and processed for immunofluorescence against GFP and mRFP. **Left:** The distribution of distances between the two loci in ~ 100 cells, binned into 0.2 µm bins. *P* values were calculated using a Wilcoxon Rank Sum Test. **Right:** the fraction of cells in which the two loci were ≤ 0.55 µm. *P* values were calculated using a Fisher Exact Test. **(A)** *INO1-TetO* vs. *URA3:INO1-LacO *or* URA3:grs1,2 INO1-LacO* grown under memory conditions. **(B) ***INO1-TetO* vs. *URA3:GRS I-LacO* under either activating or memory conditions. **(C)** *INO1-TetO* vs *URA3:GRS II-LacO* under activating or memory conditions. **(D) ***INO1-TetO *vs. *URA3:INO1-LacO put3*∆ cells under activating or memory conditions.

We previously showed that *INO1* clusters with an ectopic copy of *INO1* integrated near the *URA3* locus in a haploid cell upon recruitment to the nuclear periphery 7. To test if this clustering is maintained during memory, we compared the distances between *INO1-TetO* and *URA3:INO1-LacO* under long-term repressing conditions (overnight, +inositol) and under memory conditions (-inositol → +inositol, 3h). Under long-term repressing conditions, *INO1* and *URA3:INO1 *do not obviously cluster together, showing a broad distribution with a mean distance of 0.85 ± 0.38 µm and 20% ≤ 0.55 µm (Figure 2A). This is similar to the distribution and clustering observed for two unrelated loci in haploid nuclei 7. However, under memory conditions, there is a significant shift in the distribution to shorter distances (mean = 0.64 ± 0.33 µm;* P *= 0.0001, Wilcoxon Rank Sum test) and an increase in the fraction of cells in which the two loci are ≤ 0.55 µm (47%; *P* = 0.001, Fisher Exact test; Figure 2A). Thus, endogenous *INO1 *remains clustered with an ectopic copy of *INO1 *under memory conditions. For the experiments that follow (Figure 3, 4A), this distribution served as a control for comparison.

To assess the variance and sample size, we subjected the data to additional analysis. First, we collected three random subsamples (of 50 or 40 cells each; labeled r50 and r40 in Figure 2) from the data that were used to generate the distribution of distances under memory conditions (n = 100 cells) and compared them to the total dataset and to each other using a Wilcoxon Rank Sum test. This analysis showed that there is no significant difference between the total data and subsets of the data of ≥ 40 cells (Figure 2B).

To assess the variance between biological replicates, we performed the analysis above using total datasets (n ≥ 100 cells) or random subsamples from two independent biological replicates (Figure 2C). Of the 49 comparisons, only one (a subset of 40 compared with a subset of 50) was significantly different (*P *= 0.05; Figure 2C). In every comparison in which one of the two datasets was complete (n ≥ 100) or in which both datasets contained ≥ 50 cells, no significant differences were observed (Figure 2C). This suggests that data from ≥ 100 cells are oversampled and sufficient to avoid Type I errors (i.e. incorrect rejection of the null hypothesis that two datasets are the same).

Finally, to assess statistical power and sensitivity, we used random subsampling to compare two datasets from different conditions (repressing vs. memory conditions; Figure 3D). This analysis revealed that all sets of measurements containing ≥ 40 measurements were sufficient to reveal statistically significant difference and that the significance of the difference was greater when more cells were analyzed (Figure 3D). Therefore, comparing two datasets of 40 cells is sufficient to avoid Type II errors (failing to reject the null hypothesis that the two datasets are same), so we have measured ~ 100 cells per experiment in the work described here.

### *INO1* clustering during transcriptional memory is specific and MRS-dependent

To confirm that clustering under memory conditions is specific, we examined the positioning of *INO1* (marked with TetO) with respect to *GAL1* (marked with LacO) after simultaneously repressing both genes (Figure 3A). Both *INO1* and *GAL1* exhibit transcriptional memory, localizing at the nuclear periphery for several generations after repression 8. However, unlike *INO1* and *URA3:INO1*, *INO1* and *GAL1* did not cluster under memory conditions (Figure 3A; 15% ≤ 0.55 µm). Therefore, the clustering of *INO1* with itself under memory conditions is specific.

The MRS zip code is specifically required for localization of *INO1* at the nuclear periphery during memory and has no role in targeting of active *INO1* to the nuclear periphery 10. Mutation of the MRS disrupts peripheral localization under memory conditions. Therefore, we asked if the MRS is necessary for clustering by measuring the distances between *INO1* and *URA3:INO1* having a mutation in the MRS element (*mrs INO1*) under repressing and memory conditions (Figure 3B). Mutation of the MRS disrupted *INO1* clustering during memory (13% ≤ 0.55 µm), indicating that the clustering of *INO1* with *URA3:INO1 *after repression requires the MRS zip code.

The MRS is both necessary and sufficient to promote targeting to the nuclear periphery under memory conditions 10. To test if the MRS is sufficient to induce clustering with *INO1* during memory, we compared the positions of *INO1* and *URA3:MRS50* (a 50 bp element including the MRS; ref. 10) during memory. The MRS50 was not sufficient to induce clustering with the endogenous *INO1 *gene (Figure 3C; 6% ≤ 0.55 µm). This suggests that additional information besides the MRS is provided by the *INO1* gene to promote clustering during transcriptional memory.

### A hierarchy of DNA zip codes controls* INO1* clustering during transcriptional memory

To explore what other signals might be important for clustering of *INO1* during transcriptional memory, we tested other DNA zip codes. Targeting of active *INO1* to the nuclear periphery requires the GRS I and GRS II zip codes and mutation of both elements blocks targeting of active *INO1* to the nuclear periphery and disrupts interchromosomal clustering (Figure 1A and ref. 7). However, these mutations do not affect targeting of *INO1* to the nuclear periphery during memory, indicating that MRS-mediated targeting to the nuclear periphery is independent of GRS-mediated targeting 10. Because the MRS was necessary but not sufficient to induce clustering during memory, we hypothesized that the interchromosomal interactions of active genes might be required for the persistent clustering after repression.

To test this proposal, we examined the interaction between wild type *INO1* and *URA3:INO1* having mutations in the GRS I and GRS II (*grs1,2 INO1*). In this strain, clustering is lost under both activating 8 and memory conditions (Figure 4A; 17% ≤ 0.55 µm). Therefore, even though targeting to the nuclear periphery during transcriptional memory is GRS I- and GRS II-independent, clustering of *INO1* during memory is GRS I- and GRS II-dependent. This suggests that targeting to the nuclear periphery and interchromosomal clustering during memory must represent distinct molecular events.

Although both GRS I and GRS II are capable of mediating targeting to the nuclear periphery, GRS I directs interchromosomal clustering of *INO1* with itself and GRS II does not 8. This suggests that targeting to the periphery is necessary, but not sufficient, to promote interchromosomal clustering. Because the loss of GRS I and GRS II disrupted clustering of *INO1* during transcriptional memory, we next asked if this is due to loss of peripheral targeting under activating conditions or due to loss of interchromosomal clustering. Mutants that lack the GRS I - binding protein Put3 still target *INO1* to the nuclear periphery normally (due to GRS II function), but fail to cluster 8. We tested the effect of loss of Put3 on *INO1* clustering during memory (Figure 4B). As expected, clustering of active *INO1* with *URA3:INO1 *was disrupted in *put3*∆ mutants (Figure 4B). Consistent with the idea that clustering of active *INO1* being required for clustering of recently repressed *INO1*, loss of Put3 also disrupted clustering of *INO1* with *URA3:INO1 *during memory (Figure 4B).

This result led us to ask if the GRS I alone, which is sufficient to induce clustering with active *INO1*, or GRS II, which is not 8, could also induce clustering with *INO1* during memory conditions. Consistent with our previously published work, active *INO1* clusters strongly with *URA3:GRS I *(ref. 7; Figure 4C; 58% ≤ 0.55 µm) but not with *URA3:GRS II *(Figure 4D; 13% ≤ 0.55 µm). *INO1* did not cluster with either *URA3:GRS I* (Figure 4C; 11% ≤ 0.55 µm) or *URA3:GRS II *(Figure 4D; 15% ≤ 0.55 µm) under memory conditions. Therefore, neither the GRS I nor the GRS II are sufficient to induce clustering with *INO1* during memory and the clustering of *INO1* with *URA3:GRS I* is not stably maintained after *INO1 *repression.

### Interchromosomal clustering of *INO1* during transcriptional memory requires Nup100

The interaction of *INO1* with the NPC is different under activating and memory conditions. The interaction of the NPC with these two states requires different *cis-*acting DNA zip codes and different Nups 10. One such protein is Nup100; mutants lacking Nup100 target *INO1* to the periphery under activating conditions but not under memory conditions 10.

**Figure 5:**
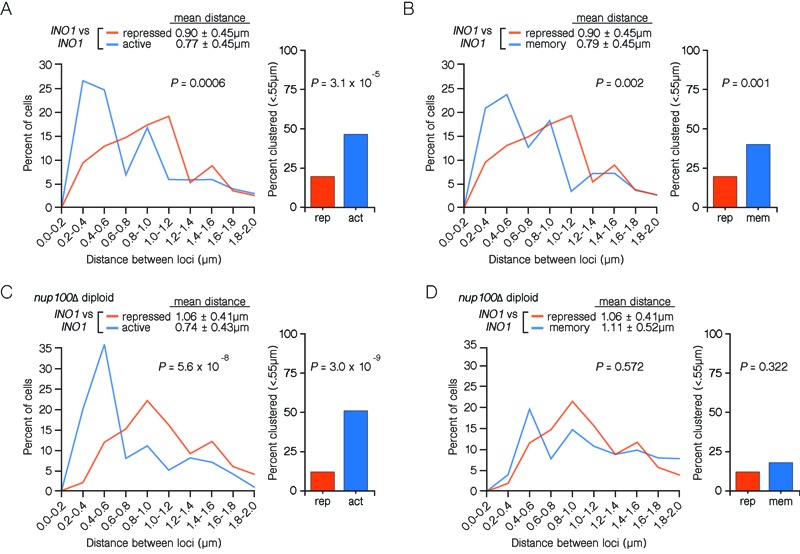
FIGURE 5: Nup100 is specifically required for *INO1* clustering during memory. Diploid cells having both alleles of *INO1* marked with the LacO array and expressing GFP-LacI were grown under repressing, activating or memory conditions. **Left:** The distribution of distances between the two loci in ~ 100 cells, binned into 0.2 µm bins. *P* values were calculated using a Wilcoxon Rank Sum Test. **Right:** the fraction of cells in which the two loci were ≤ 0.55 µm. *P* values were calculated using a Fisher Exact Test. **(A and B)** *NUP100/NUP100* diploids. **(C and D)** *nup100*∆*/nup100*∆ diploids.

To explore the role of the nuclear pore in clustering of *INO1* during transcriptional memory, we measured the distances between *INO1* alleles (marked with LacO and TetO; Figure 1B) in wild type or *nup100*∆*/nup100*∆ diploid cells grown under activating, repressing and memory conditions. Under both activating or memory conditions in the wild type (*NUP100/NUP100*) diploid cells, *INO1* clustered to very similar extent (Figures 5A and 5B). Nup100 is dispensable for clustering of active *INO1* alleles; in diploid cells lacking Nup100, the two active alleles of *INO1* clustered together (Figure 5C; mean distance = 0.74 ± 0.43 µm; 51% ≤ 0.55 µm). However, Nup100 is specifically required for *INO1* clustering during memory; under memory conditions, *INO1* clustering was lost in *nup100*∆*/nup100*∆ diploid cells (Figure 5D; mean distance = 1.11 ± 0.52 µm; 18% ≤ 0.55 µm).

### Cell cycle regulation of interchromosomal clustering of *INO1* during memory

Positioning of active *INO1*, *GAL1* and *HSP104* at the nuclear periphery is regulated through the cell cycle. Immediately after the initiation of DNA replication, all three of these genes reposition to the nucleoplasm for ~ 30 minutes before returning to the periphery during mitosis 16,17. The clustering of active *INO1* with itself is maintained during S-phase in the nucleoplasm 7. Thus, the cell cycle regulation of peripheral localization is uncoupled from interchromosomal clustering.

**Figure 6:**
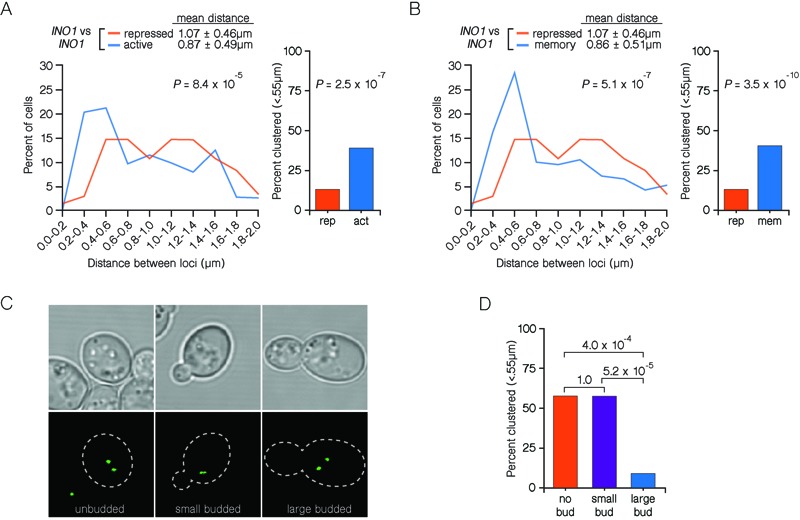
FIGURE 6: Interchromosomal clustering of recently repressed *INO1* is regulated through the cell cycle. **(A and B)** An asynchronous population of cells having *INO1:TetO* and *INO1:LacO*, expressing GFP-TetR, GFP-LacI and Pho88-mCherry (ER/nuclear envelope membrane protein) were grown under repressing, activating or memory conditions. **Left:** The distribution of distances between the two loci in ~ 100 cells, binned into 0.2 µm bins. *P* values were calculated using a Wilcoxon Rank Sum Test. **Right: **the fraction of cells in which the two loci were ≤ 0.55 µm. *P* values were calculated using a Fisher Exact Test. **(C)** Bright field (top) and green fluorescence (bottom) channels of typical cells used to measure distances in cells with different bud morphologies. The outline of the cell above is overlaid on the green channel (hatched line). **(D)** Cells were scored for both their bud morphology and the distance between the two loci and the fraction of each class of cells that was ≤ 0.55 µm was determined. *P* values were calculated using the Fisher Exact Test.

In contrast to the active genes, during transcriptional memory, *INO1* and *GAL1 *remain at the nuclear periphery throughout the cell cycle 16. Thus, it was unclear if clustering would also be maintained through the cell cycle. We tested this idea by comparing the % of unbudded (G1), small budded (S) and large budded (G2) cells in an asynchronous population in which the two alleles were ≤ 0.55 µm (Figure 6A). We utilized a diploid strain in which both copies of *INO1* were marked with LacO arrays bound to GFP-LacI (Figure 1C and 1D). This experimental setup allows rapid 3D mapping of the two alleles in live cells. However, because both spots are the same color, it is not possible to score cells in which the spots are unresolvable (i.e. only a single spot is observed), which includes some cells in which the two loci are ≤ 0.2 µm apart. Despite this limitation, we could observe clustering of *INO1* alleles under both activating (Figure 6A) and memory conditions (Figure 6B) using this system.

Distances between the two alleles of *INO1* were measured in unbudded G1, small budded S-phase and large budded G2 cells under memory conditions (Figure 6C). In unbudded cells and in small budded, we observed high level clustering (57% ≤ 0.55 µm; Figure 6D). However, clustering of *INO1* was lost in large budded cells (9% ≤ 0.55 µm; Figure 6D). In these cells, which have not yet undergone nuclear division, each allele of recently repressed *INO1* remains at the nuclear periphery 16, paired with its sister chromatid. However, the two alleles of *INO1* do not cluster together, suggesting that clustering during memory is uncoupled from gene positioning to the periphery.

## DISCUSSION

Interchromosomal clustering is a common phenomenon in eukaryotic cells. In budding yeast, as in many organisms, the 32 telomeres cluster into several foci at the nuclear periphery 18. Likewise, the ~ 250 tRNA genes, scattered throughout the genome, form two clusters, one in the nucleolus and the other near the spindle pole body 19. In flies, Polycomb repressed loci cluster together into Polycomb bodies 20,21. And in mammalian cells, co-regulated genes frequently co-localize within the nucleus 22,23,24. Thus, cells frequently utilize interchromosomal clustering as a mechanism of spatial, and perhaps transcriptional, control.

Our previous work has established another mechanism by which interchromosomal clustering is facilitated. Interaction of active yeast genes with the NPC leads to both repositioning to the nuclear periphery and clustering with other loci that share the same zip code 7. DNA zip codes are both necessary and sufficient to induce this type of interchromosomal clustering. It is unclear if clustering reflects targeting of genes to the same portion of the nuclear envelope or homotypic interactions between genes after they are targeted to an imprecise location on the envelope. Nups are essential for clustering, suggesting that targeting to the NPC is a prerequisite for clustering. However, once formed, the clusters can persist in the nucleoplasm 7. This argues either that clustering is maintained by Nups that dissociate from the NPC, as has been shown to occur in many cell types, or that the NPC serves as a site of assembly for clusters but is not required after they are assembled.

**Figure 7:**
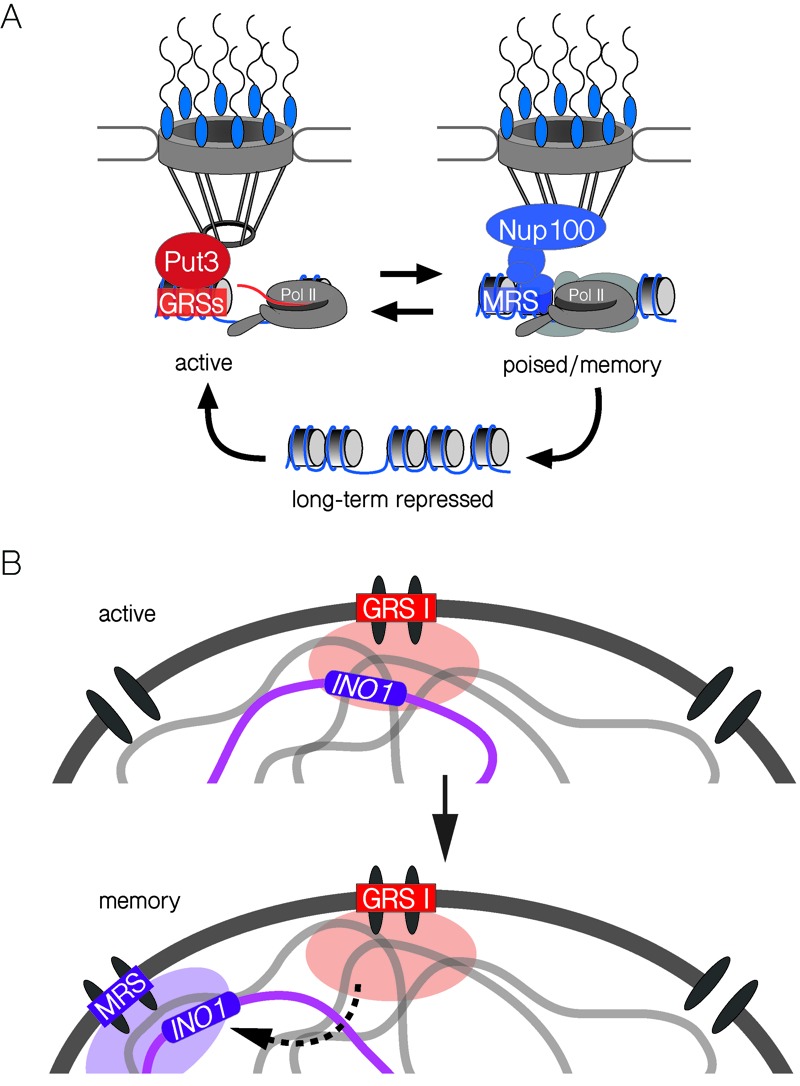
FIGURE 7: Model for zip code-dependent *INO1* targeting to the NPC and interchromosomal clustering. **(A) **The long-term repressed gene is positioned in the nucleoplasm and both the active and recently repressed memory state of the gene are positioned at the nuclear periphery through interaction with the NPC. The GRS elements control targeting to the NPC under activating conditions. The Put3 transcription factor binds the GRS I zip code and is required for GRS I-mediated peripheral targeting and interchromosomal clustering 7. The MRS element controls targeting to the NPC under memory conditions and requires Nup100 10. **(B)** The *INO1* gene clusters with other GRS I-containing loci under activating conditions (top) and this is a prerequisite for clustering with itself (and potentially other loci) in an MRS-dependent cluster for several generations after repression, during transcriptional memory.

Here we have explored a new model for interchromosomal clustering between genes that are poised for future activation. Of the genes that interact with the NPC when they are active, a small subset shows transcriptional memory, maintaining the interaction after repression (Figure 7A; ref. 8). The Nups and the DNA zip codes required for memory are distinct from those required for peripheral localization and clustering of active genes (Figure 7A; ref. 10). Here, we find that the *INO1* gene remains clustered with itself after repression. However, the molecular mechanism of clustering during memory is very different from the mechanism of clustering of active *INO1*.

Active *INO1* clusters both with itself and with other GRS I-containing genes 7. Although we cannot rule out that *INO1 *clusters with other loci during memory, this mechanism is more selective than GRS I-mediated clustering (Figure 7B). The MRS zip code is necessary, but not sufficient (when inserted at an ectopic site), to induce clustering with *INO1 *during memory. Additional information besides the MRS is required for clustering.

In addition to the MRS, the GRS zip codes are required for *INO1* clustering during memory, suggesting that clustering during memory requires previous clustering during the activating condition (Figure 7B). In other words, clustering during transcriptional memory reflects the previous clustering of active *INO1*. This is surprising because the mechanisms of targeting to the nuclear periphery when *INO1* is active or during memory are completely independent 10. Therefore, although interchromosomal clustering requires targeting to the nuclear periphery and is lost in cells in which either the zip code (MRS) or Nups (i.e. Nup100) are mutated, it has additional requirements and represents a different molecular mechanism.

Consistent with this idea, the regulation of interchromosomal clustering through the cell cycle does not perfectly mirror peripheral localization. Whereas targeting of active *INO1* and other genes is lost briefly during S-phase, peripheral localization of *INO1* during transcriptional memory is not regulated by the cell cycle 16. In contrast, the interchromosomal clustering of *INO1* alleles during memory is lost during G2. This cell cycle regulation may reflect the coordination of interchromosomal clustering with chromosome condensation and chromosome segregation.

Most of the interchromosomal clustering events described previously are associated with either silenced sites like subtelomeric regions and Polycomb repressed sites or expressed sites like tRNA genes or induced genes. Here we show that a third class of genes, those that are repressed but poised for future induction, can also cluster in the nucleus. This interaction is specific to a moment in the life history of the *INO1* gene: *INO1 *clusters with itself for 3-4 generations after repression, after which this is lost. Thus, *INO1* epigenetic transcriptional memory, which leads to heritable changes in chromatin structure and binding of poised RNA Polymerase II to the promoter, also leads to heritable interchromosomal clustering.

## MATERIALS AND METHODS

### Chemicals, yeast strains and growth conditions

Chemicals were from Sigma Aldrich, molecular biology enzymes were from New England Biolabs and yeast growth media components were from Sunrise Science Products. For experiments involving scoring peripheral localization in live cells, *PHO88* was C-terminally tagged with mCherry using PCR-based integration 25. Briefly, the *PHO88* termination codon was replaced by homologous recombination with a PCR product encoding a C-terminal translational fusion to mCherry, along with the *His5+* gene from *S. pombe*. PCR primers were designed to incorporate 45 base pairs of sequences upstream and downstream of the termination codon. All yeast strains used in this study are described in Table 1. Media, transformations and growth conditions are described in 26,27.

**Table 1:** Yeast strains used in this study.

**Strain**	**Genotype**	**Figures**
DBY326	*MAT***a*** ade2-1 can1-100 his3-11,15 leu2-3,112 trp1-1 ura3-1 INO1:TetO-Nat LEU2:TetR-GFP TRP1:LacI-RFP GAL1:p6LacO128*	Figure 3A
DBY336	*MAT***a*** ade2-1 can1-100 his3-11,15 leu2-3,112 trp1-1 ura3-1 INO1:TetO-Nat LEU2:TetR-GFP TRP1:LacI-RFP URA3:p6LacO128-INO1*	Figs 2A, 3A-C, 4A
DBY339	*MAT***a*** ade2-1 can1-100 his3-11,15 leu2-3,112 trp1-1 ura3-1 INO1:TetO-Nat LEU2:TetR-GFP TRP1:LacI-RFP URA3:p6LacOGRS_241-270_*	Figure 4C
DBY348	*MAT***a***/MATα ade2-1/ade2-1 can1-100/can1-100 his3-11,15/his3-11,15 leu2-3,112/ LEU2:TetR-GFP trp1-1/ TRP1:LacI-RFP ura3-1/URA3:p6LacO128-INO1 INO1/INO1:TetO-Nat*	Figure 3C
DBY354	*MAT***a*** ade2-1 can1-100 his3-11,15 leu2-3,112 trp1-1 ura3-1 HIS3:LacI-GFP Sec63-myc:TRP1 LEU2:LacI-GFP URA3:p6LacO128 grs1,2mtINO1:p6LacO128*	Figure 4A
DBY355	*MAT***a*** ade2-1 can1-100 his3-11,15 leu2-3,112 trp1-1 ura3-1 INO1:TetO-Nat LEU2:TetR-GFP TRP1:LacI-RFP URA3:p6LacO-mrsmtINO1*	Figure 3B
DBY362	*MAT***a***/MATα ade2-1/ade2-1 can1-100/can1-100 his3-11,15/ HIS3:LacI-GFP leu2-3,112/ LEU2:TetR-GFP trp1-1/trp1-1 ura3-1/ URA3:p6LacO128-MRS50 INO1/INO1:TetO-Nat SEC63/SEC63-13myc::Kan^R^*	Figure 3C
DBY515	*MAT***a***/MATα ade2-1/ade2-1 can1-100/can1-100 his3-11,15/ HIS3:LacI-GFP leu2-3,112/ LEU2:LacI-GFP trp1-1/trp1-1 ura3-1/ ura3-1 INO1:p6LacO128/INO1:p6LacO128*	Figure 5A and B
DBY766	*MAT***a***/MATα ade2-1/ade2-1 can1-100/can1-100 his3-11,15/his3-11,15 LEU2:TetR-GFP/LEU2:LacI-GFP trp1-1/trp1-1 ura3-1/ura3-1 PHO88/PHO88-mCherry:SpHis5^+^ INO1:TetO-Nat/INO1:p6LacO128*	Figure 6
DBY768	*MAT***a***/MATα ade2-1/ADE2 can1-100/can1-100 his3-11,15/HIS3:LacI-GFP leu2-3,112/leu2-3,112 trp1-1/TRP1:Sec63-13myc ura3-1/ura3-1 nup100∆::Kan^R^/nup100∆::Kan^R^ INO1:p6LacO128/INO1:p6LacO128*	Figure 5C and D
DBY796	*MAT***a*** ade2-1 can1-100 his3-11,15 leu2-3,112 trp1-1 ura3-1 INO1:TetO-Nat LEU2:TetR-GFP HIS3:LacI-GFP URA3:GRS II-p6LacO128*	Figure 4D
DBY798	*MAT***a*** ade2-1 can1-100 his3-11,15 leu2-3,112 trp1-1 ura3-1 INO1:TetO-Nat LEU2:TetR-GFP HIS3:LacI-GFP URA3:p6LacO128-INO1 put3∆*	Figure 4B

### Microscopy

For all experiments, cells were maintained at OD_600_ < 0.5. For inositol starvation experiments, strains were grown overnight in SDC-inositol in the presence or absence of 100 µM *myo-*inositol. To induce memory, inositol was added to 100 µM and the cells were grown for an additional 3h. Immunofluorescence microscopy was carried out as described 7,27. For live cell experiments, cells were concentrated by brief centrifugation and imaged immediately. The images were captured as 0.34 µm thick z-stacks with a Leica SP5 II Line Scanning Confocal Microscope with 100 × 1.44NA (oil immersion) objective using an Argon 488 nm and Diode Pumped Solid State 561 nm lasers in the Northwestern Biological Imaging Facility as described 28. Distances between the centers of each dot for cells in which the dots were in the same z slice were measured using LAS LiTE software.
